# Small-Angle Scattering from Nanoscale Fat Fractals

**DOI:** 10.1186/s11671-017-2147-0

**Published:** 2017-06-05

**Authors:** E. M. Anitas, A. Slyamov, R. Todoran, Z. Szakacs

**Affiliations:** 1 Joint Institute for Nuclear Research, Dubna, 141980 Russian Federation; 2 Horia Hulubei National Institute of Physics and Nuclear Engineering, Bucharest-Magurele, RO-077125 Romania; 3 Institute of Nuclear Physics, Almaty, Kazakhstan; 4 Department of Economics and Physics, Technical University of Cluj Napoca, North University Center of Baia Mare, Baia Mare, Romania

**Keywords:** 05.45.-a, 61.43.Hv, 61.05.fg, 61.05.cf, 61.43.-j

## Abstract

Small-angle scattering (of neutrons, x-ray, or light; SAS) is considered to describe the structural characteristics of deterministic nanoscale fat fractals. We show that in the case of a polydisperse fractal system, with equal probability for any orientation, one obtains the fractal dimensions and scaling factors at each structural level. This is in agreement with general results deduced in the context of small-angle scattering analysis of a system of randomly oriented, non-interacting, nano-/micro-fractals. We apply our results to a two-dimensional fat Cantor-like fractal, calculating analytic expressions for the scattering intensities and structure factors. We explain how the structural properties can be computed from experimental data and show their correlation to the variation of the scaling factor with the iteration number. The model can be used to interpret recorded experimental SAS data in the framework of fat fractals and can reveal structural properties of materials characterized by a regular law of changing of the fractal dimensions. It can describe successions of power-law decays, with arbitrary decreasing values of the scattering exponents, *and* interleaved by regions of constant intensity.

## Introduction

Many hierarchical structures generated at nano- and micro-scale have geometrical characteristics that are invariant under scale dilations, displaying self-similarity, and thus bearing fractal properties [1, 2]. Although recent advances in materials science and nanotechnology allow preparation of various artificial nano-/micro-scale deterministic fractals, with an exact self-similarity [3–7], the vast majority of natural processes generate random, statistically self-similar fractals. A good approximation in the structural studies of natural fractal formations can be done resorting to deterministic fractal models, with the same fractal dimension as of the random ones. This approach was successfully used to show that the transfer across random fractal surfaces is very close to the response of deterministic model geometries [8]. By introducing polydispersity in the construction algorithm of a deterministic fractal, similar small-angle scattering (SAS) intensities as those corresponding to random fractals can be obtained [9]. In addition, a “deterministic” approach is computationally more efficient, allowing analytic description of various properties, such as fractal form, structure factors, and the radius of gyration.

One of the most reliable methods to determine the structural properties of both deterministic and random fractals [10, 11] is employing wave diffraction in the context of small-angle scattering on nano- or micro-structured materials, using neutrons or electromagnetic waves (x-ray, light, etc.) [12]. This is why, one of the fundamental tasks in theoretical descriptions linked to experimental determinations in this research area is to reveal the relationship between the structure of fractals and their corresponding diffraction spectrum or scattering intensity distribution vs. scattering wave vector. Many experimental and theoretical studies were carried out in this direction [13–21].

Using standard theoretical computations and interpolation, the parameter that is determined from these kind of experimental measurements is the mass fractal dimension *D*_m_ (see Appendix 1), with *D*_m_<d, or surface fractal dimension *D*_s_, with *d*−1<*D*_s_<*d*. We denoted with *d* the Euclidean dimension in which the fractal is embedded. The mass fractal dimension describes the way in which the mass *M*(*r*) varies when it is embedded in a disk of radius *r*. The obtained *mass-radius* relation $M(r) \propto r^{D_{\mathrm {m}}}$ leads to the behavior of the scattering intensity $I(q) \propto q^{-D_{\mathrm {m}}}$. Evidently, *D*_m_ can be identified with the exponent of the power-law dependence of the scattering intensity as a function of the scattering wave vector *q* [18, 22]. The higher the value of *D*_m_, the more compact is the structure. Similarly, in the case of a surface fractal, its surface distribution obeys $S(r) \propto r^{2-D_{\mathrm {s}}}$, and thus, $\phantom {\dot {i}\!}I(q) \propto q^{-(2\mathrm {d}-D_{\mathrm {s}})}$ [19, 23]. Working in the three-dimensional Euclidean space, *D*_s_ approaches the minimum of two, when the surface is almost perfectly smooth. It tends to the maximum of three, if it is so folded that it almost completely fills the space.

Many experimental diffraction intensities from various chemically synthesized and biological systems are characterized, on a double logarithmic scale, by a succession of power-law decays, interleaved by regions of constant intensity. This behavior can be identified for some polymer gels [24], glycoside hydrolase for cellobiose substrate [25], polyelectrolyte complex coacervates [26], or nanoporous carbon [27]. Although the classical Beaucage model [28] can provide basic structural information about these systems (i.e., mass or surface fractal dimension and the overall size of each structural level), a more complete characterization is needed because of the large number of configurations that correspond to a fixed value of the fractal dimension. This issue was recently partially addressed by Cherny et al. [29] in the context of the small-angle scattering (SAS) models. It was shown that, for deterministic mass fractals with a single scale, additional information can be obtained, such as the fractal iteration number, the number of basic constituent units, and the scaling factor. This approach was furthermore successfully used to develop new models for fat fractals, if successions of power-law decays are present in the scattering distributions. It can be applied to structures where the overall size of the basic component units is of the same order as the distances between them [30, 31].

The theoretical model presented in this article combines previous models to extend their applicability. It describes successions of power-law decays, with arbitrarily decreasing values of the scattering exponents, *and* interleaved by regions of constant intensity. Our model is also able to provide more detailed information about each structural level in the nano-/micro-fractal. For this purpose, we consider a fat fractal, represented by a two-dimensional deterministic mass fractal with a scaling factor that is iteration number dependent, but with non-vanishing surface area in the limit of a large number of iterations, so with a positive Lebesgue measure. We derive analytic expressions of the fractal form and structure factors, and we show how to determine the fractal dimensions and scaling factors at each structural level.

## Theoretical background

Considering an array of similarly oriented, identical diffraction apertures, denoted here by *Σ*, containing *N* transparent regions, labeled by *j*, a summation over the amplitudes obtained from each aperture has to be taken into account. So, the well-known frequency distribution of the diffraction amplitude of a single aperture (Eq. (37) in Appendix 2) can be rewritten as [32]:

1 $$ A(p,s) = \sum\limits_{j=1}^{N} \iint\limits_{-\infty}^{~~~+\infty} T(x,y) e^{-2 i \pi \left(p(x+x_{j}) + s(y+y_{j})\right)}\mathrm{d}x\,\mathrm{d}y.   $$

The coordinates of a point in the local frame of the *j*th aperture are (*x*_*j*_,*y*_*j*_), and *T*(*x,y*) represents the individual transmission function corresponding to each transparent region. One can exchange summation with integration because, in our case, the apertures are described by the same individual distribution function, so that Eq. (1) can be rewritten as:

2 $$ A(p,s) = \iint\limits_{-\infty}^{~~~+\infty} T(x,y) e^{-2 i \pi (px + sy)}\mathrm{d}x\,\mathrm{d}y \times \sum\limits_{j=1}^{N}e^{ipx_{j}}e^{isy_{j}}.   $$

The integral factor from the previous equality represents the Fourier transform of the distribution function of each of the identical apertures, as noted above. This amplitude is modulated by the factor containing the summation, representing the Fourier transform of Dirac-delta distributions of the form $A_{\delta }~=~\sum _{j~=~1}^{N}(x~-~x_{j})(y~-~y_{j})$. Hence, the spatial distribution of the apertures inside the array is also accounted for. Thus, Eq. (2) can be rewritten in the form known as the array theorem [32]:

3 $$ A(p,s)~=~\mathcal{F}\left\{T(x,y)\right\} \mathcal{F}\left\{A_{\delta}\right\}.   $$

The intensity distribution of the diffracted image in the Fourier plane becomes:

4 $$ I(p,s) \equiv \left| A(p,s) \right|^{2} = \left|\mathcal{F}\left\{T(x,y)\right\}\right|^{2} \big|\mathcal{F}\left\{A_{\delta}\right\}\big|^{2}.   $$

As one expects, the first factor in the product corresponds to the scattering intensity of a single hole, while the second one reveals the way in which these holes are distributed within the diffraction aperture *Σ*. These quantities are also known as the form factor *F*(*p,q*) and, respectively, the structure factor *S*(*p,q*). This is why, the results obtained throughout the paper will be expressed using the following form of scattering intensity:

5 $$ I(p,q) \equiv F(p,s) S(p,s).   $$

## Fat fractal model and method

**Fig. 1:**
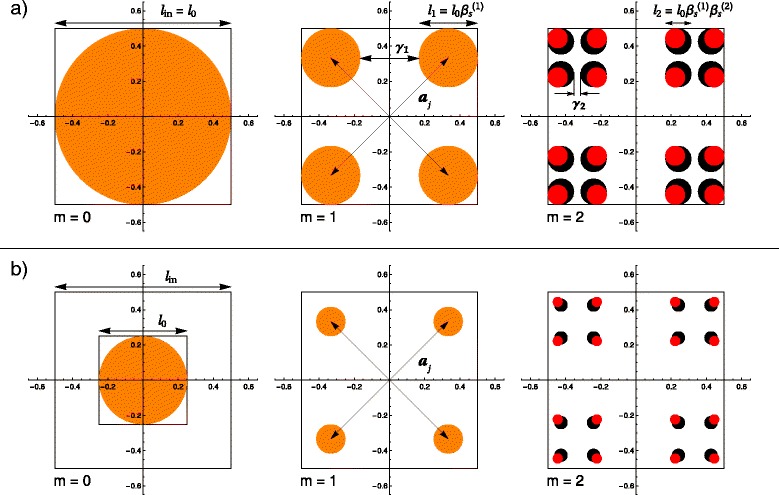
(Color online) A comparison between regular and fat fractals for the first two iterations, where the basic shape at *m* = 0 is a disk of diameter *l*_0_ and the fractal size is *l*_in_: **a** *l*_0_ = *l*_in_; **b** *l*_0_ = *l*_in_/*f*, with *f* = 2. In both cases, at *m* = 1 the structures coincide due to equal scaling factors $\beta _{\mathrm {s}}^{(1)}$. Starting with *m* = 2, the fat fractal has a bigger scaling factor $\left (\beta _{\mathrm {s}}^{(2)} > \beta _{\mathrm {s}}^{(1)}\right)$, and thus, disks have a larger diameter (*black disks*) than in the case of regular fractal (*red disks*); *a*_*j*_ are the position vectors and *γ*_*i*_ are the fractions of removed length at *i*th iteration

The detailed procedure for constructing a thin (regular) Cantor fractal is well known [33]. Only the main construction procedure is summarized here. A top to bottom approach is adopted. Starting with an initial square (or any other Euclidean shape) of edge *l*_0_ (at *m* = 0), whose center coincides with the origin of the Cartesian coordinate system and edges parallel to the coordinate system axes, any point in the square satisfies the conditions −*l*_0_/2≤*x*≤*l*_0_/2 and −*l*_0_/2≤*y*≤*l*_0_/2. At the first iteration (*m* = 1), the square is divided into four other squares, with edge length $\beta _{\mathrm {s}}^{(1)}l_{0}$. We denoted with $\beta _{\mathrm {s}}^{(1)} \equiv (1-\gamma _{1})/2$, with $0 < \beta _{\mathrm {s}}^{(1)} < 1/2$, the first iteration scaling factor, and with *γ*_1_ the fraction of the removed length at this point, as can be seen in Fig. 1 a, b) for *m* = 1. The number placed between (⋯), appearing as upper index, quantifies the iteration number. It must not be interpreted as an exponent of a power function. In terms of the scaling factor, the positions of the four squares are given by the vectors $\boldsymbol {a}_{j}~=~\left \{ \pm \beta _{\mathrm {t}}^{(1)}l_{0}, \pm \beta _{\mathrm {t}}^{(1)}l_{0}\right \}$ with all possible sign combinations, where $\beta _{\mathrm {t}}^{(1)}~=~\left (1-\beta _{\mathrm {s}}^{(1)}\right)/2$ is used to further simplify formulations. The square was chosen as an initial shape, due to the simplicity of numerical computations. Any other geometrical shape, for example a circle, can be considered. The effect of choosing another shape is observed only in the Porod region of the form factor, which is beyond the scope of this paper.

The first two steps described above are also applied in the construction of the classical version of a fat fractal, for iterations *m* = 0 and *m* = 1. This is why, up to now, these two structures coincide. To obtain the fat fractal, a modification of the algorithm used at iteration *m* = 1 must be done, by choosing another scaling factor at *m* = 2, $\beta _{\mathrm {s}}^{(2)} \equiv (1 - \gamma _{2})/2$. Applying the whole algorithm in the limit of high number of iterations [34, 35], one re-obtains the classical version of a fat fractal. It is clear from the construction that the regular version of the fractal is recovered when the scaling factors, at each iteration, are chosen to be equal $\beta _{\mathrm {s}}^{(1)}~=~\beta _{\mathrm {s}}^{(2)}~=~\cdots = \beta _{\mathrm {s}}^{(m)}$.

In order to obtain the constant plateau between two power-law decays in the behavior of SAS intensity, we have to take into account that the distances between the scattering units are much bigger than their overall size. Such an approach was firstly used in the context of surface fractal models [36, 37]. Considering the ratio *f* of the overall distance between the scattering units *l*_in_ and their overall size *l*_0_, one has:

6 $$ f~\equiv~ l_{\text{in}}/l_{0}.  $$

For scattering experiments displaying plateaus of constant intensity between two fractal regions, values of *f*≫1 should be chosen. In the case of surface fractals, increasing the value of *f* leads to a better agreement between the total SAS intensity, on the one hand, and the approximation of independent scattering units, on the other [36, 37].

**Fig. 2:**
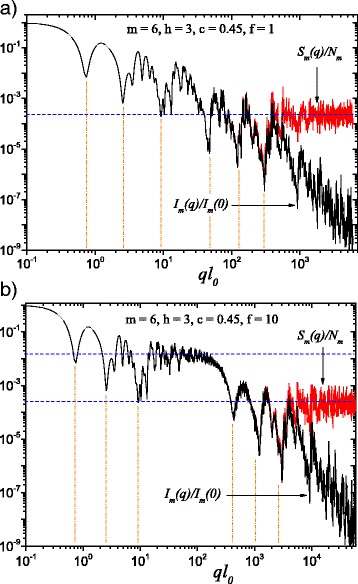
(Color online) A comparison between scattering intensity given by Eq. (22) (*black curves*) and structure factor given by Eq. (24) (*red curves*) at *m* = 6 and averaged over orientations according to Eq. (25). Here, *h* = 3 (i.e., scaling factor is kept constant for three consecutive iterations), while the basic shape in calculating the scattering intensity is a square of edge size *l*_0_: **a** *l*_0_ = *l*_in_; **b** *l*_0_ = *l*_in_/*f* (with *f* = 10). When *f*≠1, a plateau of constant intensity appears between the two generalized power-law decays (Fig. 2 b). *Horizontal lines* denote the asymptote of the structure factor ≃1/*N*_*m*_, while the minima positions are estimated according to Eq. (26)

Using the above considerations, one can describe the differences between regular and fat fractals. The influence of the factor *f*, introduced above, can also be visualized. This is why, in Fig. 1, we graphically exemplify the comparison using a disk of radius *r*_0_≡*l*_0_/2=*l*_in_/(2*f*) as our basic shape. The results of the first two iterations, displayed in each row of Fig. 1, represent the structures obtained for a regular fractal (marked by red disks) and a fat fractal (represented as a black disk), which can also be totally overlaid (marked as orange disks). In the row labeled Fig. 1 a, the factor *f* is considered to be equal to the unit so that the classical constructions and fractal shapes are obtained. The second row of the figure, denoted Fig. 1 b, exhibits the influence of the above presented factor. In these computations, we chose the arbitrary value of *f* = 2. One observes that at iterations *m* = 0 and *m* = 1, in both a and b cases, the obtained structures of the regular and fat Cantor sets are identical and completely overlaid. This is to be expected due to the common scaling factor. However, as can be seen in the last pair of images from Fig. 1, starting with *m* = 2, the radii of disks of the fat fractal are bigger because its scaling factor $\beta _{\mathrm {s}}^{(2)}$ is greater, by definition, than that of the regular one. In the last image from Fig. 2 b, the size of the disks is much smaller than in its counterpart from Fig. 2 a because of the non-unitary value of the factor *f*.

To obtain the power-laws themselves, one needs to further generalize the classical fat fractal model. This is done by considering that the scaling factor changes are not done with every single iteration, but every second, third, ⋯, or, generally speaking, every *h*th iteration. The fraction of removed lengths at the *m*th iteration is:

7 $$ \gamma_{m}~=~c^{p_{m}},   $$

with 0<*c*<1. The function *p*_*m*_ is defined as:

8 $$ p_{m} \equiv \left\lfloor 1+\frac{m-1}{h} \right\rfloor,   $$

for any positive integer value of *m*, with *h* = 1,⋯,*m*, where the floor function ⌊⋯ ⌋ was used. Thus, the scaling factor corresponding to the *m*th iteration is given by:

9 $$ \beta_{\mathrm{s}}^{(m)}~=~\frac{1-\gamma_{m}}{2}.   $$

It is clear now that the purpose of the function *p*_*m*_ is to keep the scaling factor constant for *h* iterations (*h*<*m*).

The components of position vectors of each square can be written as:

10 $$ \beta_{\mathrm{t}}^{(m)} = \frac{\beta_{\mathrm{s}}^{(m)}}{2} + \frac{\gamma_{m}}{2},   $$

while the edge length of each square is given by:

11 $$ l_{m}=\frac{l_{0}}{2^{m}}\prod_{i=1}^{m}(1-\gamma_{i}).   $$

The factor *f* is to be used in the formula of the length *l*_0_ to take into account that for iterations between the (*h*+1)th and *m*th, the size of squares decreases with respect to the distances between them:

12 $$  l_{0} = \left\{\begin{array}{ll} l_{\text{in}}, & \mathrm{for~~iterations~~} \leq h \\ l_{\text{in}}/f, & \mathrm{for~~iterations~~} > h, \end{array}\right.  $$

where *h* < *m*. The number of squares at each iteration is:

13 $$ N_{m}~=~4^{m}.   $$

Thus, at every scale, considered as iteration with constant scaling factor, one has a different fractal dimension given by [29, 38, 39]:

14 $$ D_{\mathrm{m}}~=~-\frac{2 \ln 2}{\ln \beta_{\mathrm{s}}^{(m)}}.   $$

In the limit of a big number of iterations, the fractal dimension of the constructed fractal set will be [34]:

15 $$ D \equiv \lim\limits_{m \rightarrow \infty}{\frac{\ln N_{m}}{\ln (l_{0}/l_{m})}} = 2,   $$

which is the expected value for a two-dimensional fat fractal. Finally, if *a*_*i*_ is the relative area removed at *i*th iteration, then $\prod _{i=1}^{m}(1-a_{i}) > 0$ if $\sum _{i=1}^{\infty } a_{i}< \infty $, and thus, the model satisfies the definition and characteristics of fat fractals [35].

## Results and discussion

According to the Babinet principle, we can conclude that at *m*th iteration, the apertures in the grating are the remaining squares in the fractal, while the removed parts become opaque to the radiation.

### Monodisperse scattering intensity and structure factor

In order to derive the analytic expression of the scattering intensity for the fat Cantor fractal, we start by writing the recurrence relation of the grating transmittance for an arbitrarily iteration corresponding to 1*D* case. At *m* = 0, we have

16 $$ T_{0}(l_{0}, x) \equiv \text{rect}(l_{0}, x) =\left\{\begin{array}{ll} 1, & |x| < l_{0}/2 \\ 1/2, & |x| = l_{0}/2, \\ 0, & \text{otherwise}. \end{array}\right.   $$

Taking into account the construction algorithm of the fractal, the transmittance after the first iteration is given by:

17 $$  \begin{aligned} T_{1}(l_{1}, x,) = T_{0}(l_{1}, x) \ast \delta\left(\frac{x-l_{0}\beta_{\mathrm{t}}^{(1)}}{l_{1}}\right) + \\ T_{0}(l_{1}, x) \ast \delta\left(\frac{x+l_{0}\beta_{\mathrm{t}}^{(1)}}{l_{1}}\right),~~~~~~~~~~~~ \end{aligned}  $$

where *δ*(*x*−*a*) is the one dimensional Dirac-delta distribution at *x*=*a*. The symbol ∗ represents the convolution operator. Hence, at *m*th iteration, we can write:

18 $$  \begin{aligned} T_{m}(l_{m}, x) = T_{m-1}(l_{m}, x) \ast \delta\left(\frac{x-u_{m}}{l_{m}} \right) + \\ T_{m-1}(l_{m}, x, y) \ast \delta\left(\frac{x+u_{m}}{l_{m}} \right),~~~~~~~~~~~~ \end{aligned}  $$

where $u_{m}~=~l_{0}\beta _{\mathrm {t}}^{(m)}\prod _{j=1}^{m-1}\beta _{\mathrm {s}}^{(j)}$. Performing a Fourier transform on Eq. (18), one finds that the scattered amplitude at *m*th iteration is:

19 $$ A_{m}(p)=2^{m}\frac{\sin(\pi p l_{m})}{\pi p l_{m}}\prod\limits_{i=1}^{m}\cos(2\pi p u_{i}).   $$

Since the 2*D* fat fractal model is a direct product of two one-dimensional fat fractals, its Fourier transform can be written as a product of two one-dimensional Fourier transforms. Hence, the two-dimensional scattering amplitude can be written as:

20 $$ A_{m}(p,s)\equiv A_{m}(p) A_{m}(s),   $$

and thus,

21 $$  \begin{aligned} A_{m}(p, s) = N_{m}\frac{\sin(\pi p l_{m})}{\pi p l_{m}}\frac{\sin(\pi s l_{m})}{\pi s l_{m}} \times \\ \prod\limits_{i=1}^{m}\cos(2\pi p u_{i})\cos(2\pi s u_{i}), \end{aligned}  $$

so that the scattering intensity becomes:

22 $$  \begin{aligned} I_{m}(p, s) =\left(\frac{\sin(\pi p l_{m})}{\pi p l_{m}}\frac{\sin(\pi s l_{m})}{\pi s l_{m}} \right)^{2} \times \\ N_{m}^{2} \left(\prod\limits_{i=1}^{m}\cos(2\pi p u_{i})\cos(2\pi s u_{i}) \right)^{2}. \end{aligned}  $$

The first factor in the previous equation, representing the diffraction intensity due to the form factor, as stated in Eq. (5):

23 $$ F_{m}(p, s) = \left(\frac{\sin(\pi p l_{m})}{\pi p l_{m}}\frac{\sin(\pi s l_{m})}{\pi s l_{m}} \right)^{2},   $$

corresponds to scattering intensity obtained from a single square of edge *l*_*m*_. The second factor, representing the diffraction intensity due the structure factor, as stated in Eq. (5):

24 $$ S_{m}(p, s) = N_{m}^{2}\left(\prod\limits_{i=1}^{m}\cos(2\pi p u_{i})\cos(2\pi s u_{i}) \right)^{2},   $$

describes the way in which squares are distributed. The total scattered radiation intensity is the product of *F*_*m*_(*p,s*) and *S*_*m*_(*p,s*).

The power-law decay of the intensity, as formulated in Eq. (22), is obtained after performing the average over all orientations [29]. Considering equal probability for any orientation, the average can be calculated in the case of two-dimensional fractals by integrating over all directions of the scattering vector ***q*** = (*p,s*):

25 $$ \langle f(p, s) \rangle = \frac{1}{2\pi} \int_{0}^{2\pi}f(q,\phi)\mathrm{d}\phi,   $$

where *p* = *q* cos*ϕ* and *s* = *q* sin*ϕ*. Thus, the scattering intensity *I*(*q*) is obtained as a function of the modulus of momentum transfer *q*≡|***q***|.

Because, from the definition of structure factor, one has $S_{m}(0)~=~N_{m}^{2}$, where *N*_*m*_ is the number of squares, as defined in Eq. (13), the standard procedure of normalization *S*_*m*_(0) = 1 can be adopted, as described in [11, 29].

The results computed for the monodisperse scattering intensity *I*_*m*_(*q*) and structure factor *S*_*m*_(*q*), with *m* = 6, are displayed in Fig. 2 for the classical fat fractal (*f* = 1 in Fig. 2 a) and, for the extended fat fractal model developed in this work (*f* = 10 in Fig. 2 b). To obtain the Fig. 2 b, we considered *h* = 3 so that the scaling factor $\beta _{\mathrm {s}}^{(1)}$ of the first three iterations was kept constant, then it had an other constant value $\beta _{\mathrm {s}}^{(2)}$ for next three iterations. As expected, in both cases (for *f* = 1 and *f* = 10), the differences between scattering intensity on the one hand, and the structure factor on the other, can be observed when $q \gtrsim 1/l_{m}$. In this region, the scattering intensity has a power-law decay *I*(*q*)∝*q* ^−3^. The structure factor has an asymptotic value which tends to 1/*N*_*m*_, represented by the horizontal line in Fig. 2 a or the lower horizontal line in Fig. 2 b [29, 33].

A succession of two generalized power-law decays, identifiable as a superposition of maxima and minima, over a simple power-law decay, can be seen in Fig. 2 a. But in Fig. 2 b, a region of approximately constant intensity, in the domain 20≲*ql*_0_≲100, can be clearly distinguished, encompassed by the two successive generalized power-law decays. This is due to the decrease in the size of the squares by one order of magnitude (*f* = 10) compared to the distances between them. This region, observable around the upper horizontal line in Fig. 2 b has the asymptote 1/*N*_3_, the same as the one of the classical fat fractal’s structure factor, displaying a behavior similar to the case of considering only the first three iterations.

In addition, it can be seen in Fig. 2 that the number of minima at each scale coincides with the number of constant scaling factor iterations. These minima occur when the radiation passing through different squares inside the fractal interferes and are in phase opposition, and thus, the most frequently encountered distances between the center of squares (2*u*_*m*_) are equal with *π*/*q*. This is why, the approximate positions of the minima are obtained from the relation:

26 $$ q_{i} \simeq \frac{\pi}{2 u_{i}},~~~~i=1, \cdots, m   $$

indicated in the Fig. 2 by vertical lines. For the first six iterations, one observes a quite good agreement between the positions computed using Eq. (26), and those found in the scattering intensity, or structure factor. This approximation could be less accurate for higher iterations, once the iteration number increases above a certain value because in these cases, more and more distances are comparable to the most frequently encountered one. Nevertheless, this approximation shall work fairly well in practice, where one can hardly expect to distinguish more than four or five such minima.

**Fig. 3:**
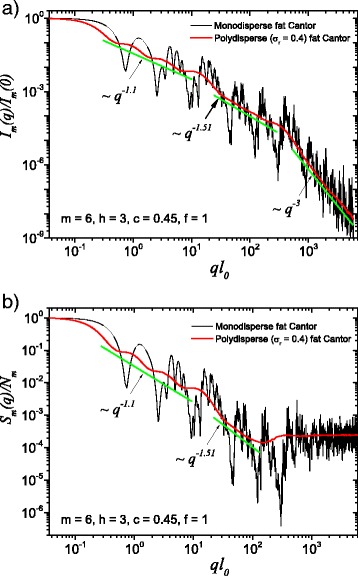
(Color online) A comparison between monodisperse and polydisperse systems: **a** scattering intensity (Eq. (22)); **b** structure factor (Eq. (24)), averaged over all orientations of the fractal, according to Eq. (25). Here, *f* = 1, *m* = 6, *h* = 3 (i.e., the scaling factor is kept constant for three consecutive iterations), and the basic shape is a square of initial edge length *l*_0_ = *l*_in_. For both cases, the polydispersity smears out monodisperse scattering curves, and the fractal dimensions can be recovered at each structural level

For each individual scale, in a given range 1/(2*u*_*i*_)≲*q*≲1/(2*u*_*i*+1_), the diffraction pattern is produced by the interference of only the *i*th fractal iteration. This can be used to show that, within this interval, the functions *I*_*m*_(*q*)*q* ^*D*^ and *S*_*m*_(*q*)*q* ^*D*^ are log-periodic [29], where *D* is the fractal dimension corresponding to a given scale. In particular, for the results shown in Figs. 2 and 3, the functions *I*_*m*_(*q*)*q* ^−1.1^ and *S*_*m*_(*q*)*q* ^−1.1^ are log-periodic with the period $1/\beta _{\mathrm {s}}^{(1)}$ for the first three iterations, while *I*_*m*_(*q*)*q* ^−1.51^ and *S*_*m*_(*q*)*q* ^−1.51^ are log-periodic with $1/\beta _{\mathrm {s}}^{(2)}$ for the second group of three iterations.

In a similar manner to deterministic mass fractals, Eq. (26) can be used to obtain several structural parameters characterizing fat fractals. First, the total number of minima coincides with the total number of fractal iterations. Figure 2 shows that the fractal consists of three iterations with scaling factor $\beta _{\mathrm {s}}^{(1)}$ and three iterations with scaling factor $\beta _{\mathrm {s}}^{(2)}$. Second, from the periodicity of these minima (or from the periodicity of *I*_*m*_(*q*)*q* ^*D*^ and *S*_*m*_(*q*)*q* ^*D*^), the scaling factors can be recovered. In Fig. 2 b, the scaling factor $\beta _{\mathrm {s}}^{(1)}$ can be obtained from the periodicity of minima at *ql*_0_≃7,25, and 90, while the scaling factor $\beta _{\mathrm {s}}^{(2)}$ can be obtained from the periodicity of minima at *ql*_0_≃400,1000 and 2500. In addition, the length of the intermediate plateau between fractal regions can be used as an indication of the ratio (*f*) of distances between scattering units, and their overall size. In Fig. 2 b, this range corresponds to 13≲*ql*_0_≲130.

### Polydisperse scattering intensity and structure factor

In this part of our work, we can consider now that the grating sizes obey a distribution function *D*_N_(*l*_0_), defined in such a way that *D*_N_(*l*_0_)d*l*_0_ gives the probability of the size of the fractal grating to be in the interval (*l*_0_,*l*_0_+d*l*_0_). This step introduces polydispersity in our fat fractal model. We exemplify this by choosing a log-normal distribution:

27 $$ D_{\mathrm{N}}(l_{0}) = \frac{1}{\sigma l_{0} (2\pi)^{1/2}}e^{-\frac{\left(\log(l_{0}/\mu)+\sigma^{2}/2\right)^{2}}{2\sigma^{2}}},   $$

with relative variance $\sigma _{\mathrm {r}} = \left (\left \langle l_{0}^{2} \right \rangle _{D} - \mu ^{2}\right)^{1/2}/\mu $, mean value *μ*=〈*l*_0_〉_*D*_, and variance $\sigma = \left (\log \left (1+\sigma _{\mathrm {r}}^{2}\right)\right)^{1/2}$. Using Eqs. (21) and (27) one obtains the polydisperse intensity averaged over the distribution function:



where  is the corresponding area at *m*th iteration. The structure factor is calculated in a similar manner, but without the term  [29].

**Fig. 4:**
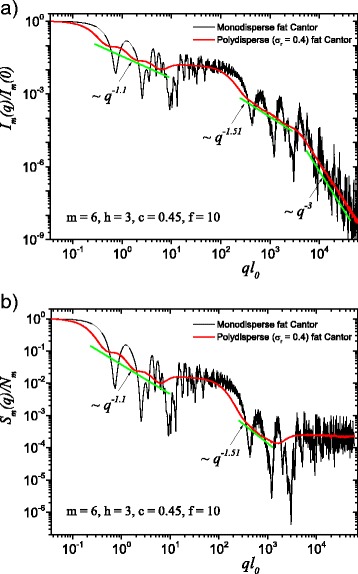
(Color online) A comparison between monodisperse and polydisperse systems: **a** scattering intensity (Eq. (22)); **b** structure factor (Eq. (24)), averaged over all orientations of the fractal, according to Eq. (25). Here, *f* = 10 (and thus, a region of constant intensity appears at about 20≲*ql*_0_≲100), *m* = 6, *h* = 3 (i.e., the scaling factor is kept constant for three consecutive iterations), and the basic shape is a square of initial edge length *l*_0_ = *l*_in_. For both cases, the polydispersity smears out monodisperse scattering curves, and the fractal dimensions can be recovered at each structural level

The computed results in the case of polydisperse (red curves) and monodisperse (black curves) scattering intensities (labeled by a) and structure factors (labeled by b) can be seen in Figs. 3 and 4. The difference between them is given by the value of the *f* factor. In Fig. 3, the classical construction of a fat fractal was used so that *f* = 1, while taking into account the smaller sizes of the basic units leads to the choice of *f* = 10 in Fig. 4. Polydispersity is calculated for a relative variance of *σ*_r_ = 0.4. It can be seen that the oscillations are smeared out, the overall amplitude decreases, so that the scattering curves become smoother [29, 40]. However, for this particular value of *σ*_r_, the positions of main minima and maxima are still observable.

More generally, for small values of *σ*_r_ (i.e., small enough that the oscillations are observable), the estimation given by Eq. (26) can be still used. Hence, the number of fractal iterations, the scaling factor at each structural level, the ratio of the distances between scattering units, and their overall size can be recovered. When *σ*_r_ is increased to high enough values so that oscillations are completely smeared out, the scattering curves become simple power-law decays. Since we used a narrow bell-shaped distribution, the scattering exponent is preserved. Moreover, it gives, for each power-law decay, the fractal dimension of that particular structural level. This is in good agreement with the theoretical estimation of Eq. (14). This is also in accordance with experimental setups, where almost every scattering curve has a certain degree of polydispersity. Thus, our developed fat fractal model, with an interleaved region of constant intensity, recovers the fractal dimension at each structural level from polydisperse experimental data.

## Conclusions

In this article, we suggest a theoretical model that generalizes the standard one for nanoscale fat fractals. It is characterized by the fact that the initial edge size of the elementary unit shape is taken to be much smaller than that of the overall size of the fractal, and thus, much smaller than the distances between the elementary units inside the fractal. Figure 1 b illustrates the basic model, when a quotient of 1/2 is considered in-between these quantities, respectively.

Based on this model, an analytical formula is calculated and presented, in Eq. (22) for the scattering intensity and in Eq. (24) for the structure factor. Averaging over all possible orientations is done according to Eq. (25). These averaged quantities are characterized, on a double logarithmic scale, by the presence of two structural levels, and thus by two power-law decays interleaved by a region of constant intensity, represented by a plateau, as seen in Figs. 2 b and 4. This plateau coincides with the asymptotic region of the structure factor of the fat fractal, as if we would have considered only the contribution from the first structural level, when the scaling factor was kept constant. The asymptotic values of the plateaus can be used to obtain the number of scattering units for each structural level. The length of the plateau is controlled by the value of *f*. The power-law decays encompassing the plateau are obtained by keeping constant the scaling factors for a finite number of iterations, in our case, as an example, for three out of a total of six. The slope of the second power-law decay is higher because the values of scaling factors, by definition, increase at each structural level, and this is confirmed by our numerical computations, as can be seen in Figs. 2, 3, and 4.

We also described the polydisperse case of the fat fractal model. Here, the sizes of the composing units obey, as an example, a log-normal distribution function. We obtained smoothed curves for the scattering intensities and structure factors. The monodisperse scattering curves as well as the polydisperse ones, with small enough values of the relative variance, allow to obtain the *scaling factors* at each structural level, while the scattering exponents in the polydisperse curve give the *fractal dimensions* at each structural level. The chosen value of 0.4 for the relative variance is meant to illustrate the case in which one can still observe some minima in the scattering characteristics, and the curves still retain a shape close to power-law decays.

The results obtained in the framework of the suggested model can be used to reveal structural properties of fractal materials characterized by a regular law of changing of the fractal dimensions. The proposed model is also a very versatile one because it can be extended to include other features such as different shapes of the elementary unit, more than two structural levels, or it can be adapted to work in other Euclidean dimensions. These results are useful for a detailed description of experimental diffraction data in the context of small-angle scattering obtained from various complex nano- and micro- scaled hierarchical structures.

## Appendix

### fractal dimension

Mass and, respectively, surface fractal dimensions are probably the most important quantities that characterize a fractal. Actually, we will deal only with deterministic mass fractals, and we shall refer to mass fractal dimension, simply as the fractal dimension (*D*_m_).

In general terms, the *mass-radius* relation can be rewritten as [2]:

29 $$ M(r) = A(r) r^{D_{\mathrm{m}}},   $$

where the scaling law correction *A*(*r*) tends to a constant value if *r*→*∞*.

If it is known a priori that the structure is a fractal in the high number limit, the fractal dimension can be found straight from the first iteration. To illustrate this procedure, let us consider a fractal of size *l*_0_, composed of *k* elementary units at the first iteration, each of size *β*_s_ *l*_0_, where *β*_s_ is a scaling factor. Since the *mass-radius* relation, given by Eq. (29), is equivalent with the *scale-invariance* relation [2]:

30 $$ M(\beta_{\mathrm{s}}l_{0}) = \beta_{\mathrm{s}}^{D_{\mathrm{m}}}M(l_{0}),   $$

one can write *M*(*l*_0_)=*kM*(*β*_s_ *l*_0_). Using Eq. (29), one obtains a direct method to compute the fractal dimension, via:

31 $$ k \beta_{\mathrm{s}}^{D_{\mathrm{m}}} = 1.   $$

### fraunhofer diffraction and the array theorem

Let us consider a two-dimensional diffracting aperture *Σ*, laid in the (*x,y*) plane, illuminated in the positive *z* direction. In an observation plane (*u,v*), parallel to *Σ*, the complex-valued amplitude of the obtained diffraction image, computed using the framework of scalar theory of diffraction, according to the Huygens-Fresnel principle, can be written as [41]:

32 $$ A(u,v) = \frac{z}{i\lambda} \iint\limits_{\Sigma} A(x,y)\frac{e^{ikr}}{r^{2}} \mathrm{d} x\,\mathrm{d} y.   $$

In the previous formula, $r = \sqrt {z^{2}+(u-x)^{2}+(v-y)^{2}}$ is the distance between two arbitrarily points taken, respectively, from the plane containing *Σ* and from the observation plane. For the Fraunhofer diffraction model to be applicable, this distance must satisfy the condition of being much bigger than the wavelength *λ*.

Performing a binomial expansion of the square root in Eq. (32) and retaining only the first two terms, one obtains [41]:

33 $$ r \approx z\left(1 + \frac{(u-x)^{2}}{2z^{2}} + \frac{(v-y)^{2}}{2z^{2}}\right).   $$

This approximation leads to the Fresnel diffraction integral:

34 $$ \frac{A(u,v)}{P(u,v)} = \iint\limits_{-\infty}^{~~~+\infty} \left\{A(x,y) e^{i\frac{k}{2z}(x^{2} + y^{2})}\right\} e^{-i \frac{2\pi}{\lambda z}(ux + vy)}\mathrm{d}x\,\mathrm{d}y,   $$

where the prefactor *P*(*u,v*) is given by

35 $$ P(u,v) = \frac{e^{ikz}e^{i\frac{k}{2z}(u^{2}+v^{2})}}{i\lambda z},   $$

and *k*=2*π*/*λ*. Considering, in addition, that the condition *z*≫*k* Max(*x* ^2^+*y* ^2^)/2 is satisfied, one has $\text {Exp}{\left (\frac {k}{2z}(x^{2}+y^{2})\right)} \simeq 1$. Rewriting Eq. (34), the Fraunhofer approximation becomes:

36 $$ A(u,v) = P(u,v)\iint\limits_{-\infty}^{~~~+\infty} A(x,y) e^{-i \frac{2\pi}{\lambda z}(ux + vy)}\mathrm{d}x\,\mathrm{d}y.   $$

Denoting the spatial frequencies with *p*=*u*/(*λ* *z*) and *s*=*v*/(*λ* *z*) and ignoring the multiplicative phase factor *P*(*u,v*) preceding the integral in Eq. (36), the amplitude becomes simply the Fourier transform of the distribution of the *Σ* aperture. Considering that the illumination is made using a monochromatic, unit-amplitude plane-wave, at normal incidence, and that the field distribution across the aperture is equal to its transmission function *T*(*x,y*), one obtains the frequency distribution of the diffraction amplitude in the phase space:

37 $$ A(p,s) = \iint\limits_{-\infty}^{~~~+\infty} T(x,y) e^{-2 i \pi (px + sy)}\mathrm{d}x\,\mathrm{d}y.   $$
